# A Fall in Infant Mortality

**Published:** 1917-06-16

**Authors:** R. Veitch Clark

**Affiliations:** Medical Officer of Health. Public Health Department, Town Hall, Croydon


### A Fall in Infant Mortality.

*To the Editor of* THE HOSPITAL.

SIR,—My attention has been directed to a reference in your paper to the offer made by the Mayor of Croydon of a War Savings Certificate to each baby born during Baby Week, and your comment upon it that the money would be better spent if it were awarded to those cases where the babies are alive after twelve months. I write to point out that the information which you have evidently received is incomplete. The offer made by the Mayor is contingent upon the regular attendance by the mother with the infant at one of the Baby Centres in the town for a period of at least six months after the birth of the child. The offer is an extremely generous one, and I think ought to be recognised fully as such, as the sole aim of the Mayor in making it is to stimulate the interest of the inhabitants of the Borough in the welfare of mothers and infants. The condition which the Mayor has made is in many respects better than that suggested in your paper, as it not only stimulates individual interest in the cases themselves but directs attention to the baby work which is being carried on with a view to the saving of child welfare generally.

May I request the courtesy of publication of this letter in your journal?—Yours faithfully,

R. VEITCH CLARK,
Medical Officer of Health.

Public Health Department, Town Hall,
Croydon, June 11, 1917.

[The Mayor of Croydon's action has our warm sympathy, and we are indebted to Dr. Clark for the interesting particulars he has sent us.—ED. THE HOSPITAL.]

